# *Astragalus* Polysaccharide RAP Induces Macrophage Phenotype Polarization to M1 via the Notch Signaling Pathway

**DOI:** 10.3390/molecules24102016

**Published:** 2019-05-27

**Authors:** Wei Wei, Zhi-Peng Li, Zhao-Xiang Bian, Quan-Bin Han

**Affiliations:** School of Chinese Medicine, Hong Kong Baptist University, 7 Baptist University Road, Kowloon Tong, Hong Kong, China; 12467243@life.hkbu.edu.hk (W.W.); lizhipeng0903@163.com (Z.-P.L.); bzxiang@hkbu.edu.hk (Z.-X.B.)

**Keywords:** Astragali Radix, tumoricidal responses, M1 markers, Notch signaling, macrophages, polysacchairdes

## Abstract

Macrophages occur in polarized phenotypes, whose characteristics determine the role they play in tumor growth. The M1 phenotype macrophages promote tumoricidal responses and suppress tumor growth. Our previous study showed that a polysaccharide isolated from Radix Astragali, named RAP, was itself non-cytotoxic but induced RAW264.7 cells’ cytotoxicity against cancer cells. The current study was undertaken to determine its mechanism. Series studies was conducted to show that RAP is able to induce much higher gene expression of M1 markers, including iNOS, IL-6, TNF-a, and CXCL10, compared with the control group. When RAP-induced BMDMs were transplanted together with 4T1 tumor cells in BALB/c mice, both tumor volume and tumor weight decreased. Further studies indicated that RAP induces the Notch signaling pathway in RAW264.7 cells. The function of Notch signaling in macrophage polarization was confirmed by using γ-secretase inhibitor. These results suggested that *Astragalus* polysaccharide RAP induces macrophage’s polarization to M1 phenotype via the Notch signaling pathway.

## 1. Introduction

*Astragalus* polysaccharides, which comprise the majority of the chemical components of Astragali Radix decoction, have been shown to have diverse bio-activities, including immunomodulatory [1,2], antioxidant [3,4,5], antitumor, anti-diabetic [6,7], and anti-inflammatory activities [8,9]. Both clinical trials and animal studies have shown that *Astragalus* polysaccharide has an anti-tumor effect [10,11]. Several studies have demonstrated that the anti-tumor effect appears to be related to its immune system-modulating activities [12,13,14].

In our previous study, a water-soluble polysaccharide named RAP was purified from the water extract of Astragali Radix. It was found to be able to stimulate human mononuclear cells to secrete IL-1β, IL-10, TNF-α, GM-CSF, and IL-12p40 [15]. RAP itself failed to exhibit any cytotoxicity against 4T1 cells, but RAP significantly enhanced the cytotoxicity of the supernatant of RAW264.7 cells on 4T1 cells. These results suggest that RAP’s anti-tumor effects are associated with their immunomodulating effects on macrophages [16]. However, the precise molecular mechanisms of its antitumor effect have not been clearly elucidated.

The phenotypes of macrophages might explain a part of the mechanism of action. A recent study demonstrated that polysaccharides from the mushroom Agaricus blazei Murrill reverses phenotypes of myeloid derived suppressor cells (MDSCs) from M2 to M1, which in turn suppress tumor cell growth [17]. As the major population of MDSCs, macrophages also have two extreme representative phenotypes, designated M1 and M2 [18,19]. When appropriately stimulated, macrophages assume the M1 phenotype and have the potential to kill tumor cells. In the tumor micro-environment, however, differentiating macrophages are classically polarized by anti-inflammatory molecules into the M2 phenotype [20]. These M2 type tumor-associated macrophages (TAMs) promote tumor cell survival, proliferation, and dissemination [21,22]. High levels of TAMs are often correlated with a bad prognosis, e.g., metastasis [23,24]. Therefore, macrophages are an important drug target for cancer therapy.

It has been known that the Notch signaling pathway plays an important role in macrophage polarization. Activation of Notch signaling induces the macrophages’ phenotype polarization from M2 to M1 and increases their antitumor capabilities [25,26]. Macrophages deficient in canonical Notch signaling typically show M2 phenotypes. Activated Notch1 and expression of the Notch target genes Hes1 and Deltex significantly modulate expression of TNF-α, IL-6, and IL-10, through activation of NF-κB. Furthermore, the Notch signaling pathway is closely associated with activation of TLRs’ signaling in that stimulation of TLRs up-regulates Notch1 and Notch2 gene expression in macrophages [27,28]. Regulation of inflammatory responses depends on the Notch signaling activation induced by LPS through the JNK-dependent pathway [29]. Our previous study showed that RAP induced TNF-α, IL-6, and NO production through TLR4 receptor, JNK and NF-κB signaling pathway in macrophage-like cell line RAW264.7 cells [16]. Therefore, in this study, we aim to find if RAP induces antitumor activity by reversing macrophage polarization to M1 through the Notch signaling pathway in vitro.

## 2. Results

### 2.1. RAP-Stimulated BMDMs Decreased Tumor Volume and Tumor Weight

**Figure 1:**
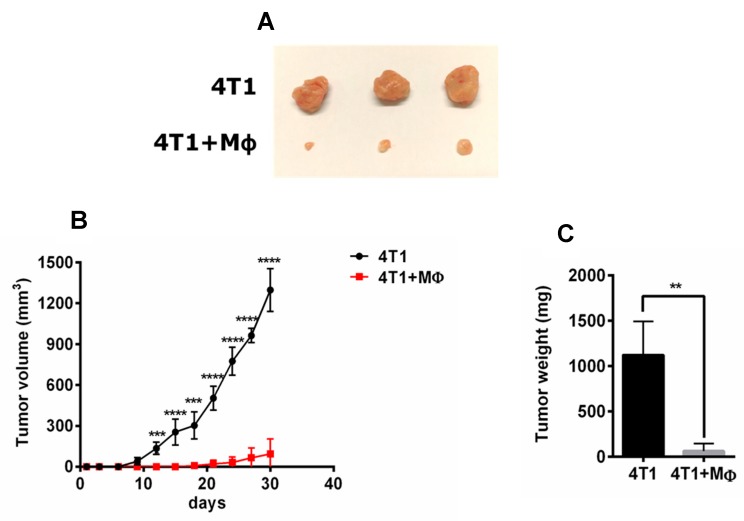
Tumoricidal effect of RAP-stimulated BMDMs in a 4T1-induced animal model. The BALB/c mice (3 mice/group) were injected with 4T1 cells and 4T1 cells plus BMDMs stimulated with RAP in mammary grand fat pad. Tumor volumes were measured every 3 days (**B**), **** *p* < 0.0001, *** *p* < 0.001, ** *p* < 0.01; The tumor weight (**C**) and tumor image (**A**) were shown as indicated. A significant difference of tumor weight was observed by one-way ANOVA test at the 30th day (** *p* < 0.01) (**C**).

After 30 days, tumor volume and tumor weight of the 4T1 cells plus RAP-stimulated BMDMs group were significantly lower than those of the 4T1 cell injection alone group (*p* < 0.01), as shown in Figure 1. Figure 1A clearly shows that the tumors of the 4T1 cells plus RAP-stimulated BMDMs injection group are much smaller than those of the 4T1 cells injection group. On the 30th day, the tumor volume of the 4T1 cells plus RAP-stimulated BMDMs injection group was 1297.19 ± 156.86 mm^3^, but the tumor volume of the 4T1 cells injection group was only 94.62 ± 108.76 mm^3^. Consistent with the differences in tumor volume, the tumor weight of the 4T1 cells plus RAP-stimulated BMDMs injection group (64.93 ± 80.59 mg) was much lower than that of the 4T1 cell injection group (1123.05 ± 369.88 mg, Figure 1C). The repeat experiments for this assay were listed in the Supplementary Information Figure S1. These results showed that macrophages (BMDMs) stimulated by RAP could significantly delay tumor growth of 4T1-bearing mice, suggesting that the RAP-induced macrophages are M1 phenotype macrophages.

### 2.2. Morphology of BMDMs Induced by RAP

After incubation for 7 days, presence of a surface marker on bone marrow cells was confirmed by APC-F4/80 (Biolegend, San Diego, CA, USA) staining, and then analyzed by FCM. As shown in Supplementary Information Figure S2A, more than 98% of the adherent cells were bone marrow-derived macrophages (BMDMs) highly expressing F4/80 surface marker. After 20 ng/mL of IL-4 stimulation for 24 h, the BMDMs showed to be M2 phenotype because they highly expressed CD206 compared to the control cells (Supplementary Information Figure S2B). The BMDMs showed M1 phenotype when cells were treated with 1 μg/mL of LPS for 24 h. Therefore, we used cells treated with LPS as the positive control group.

**Figure 2:**
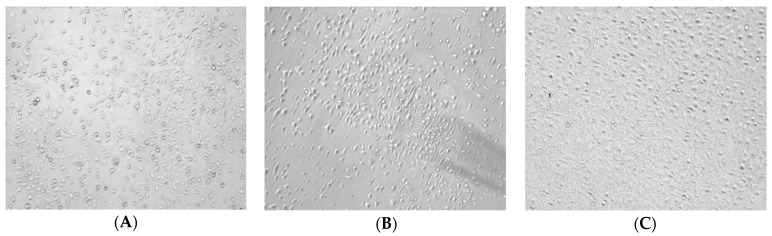

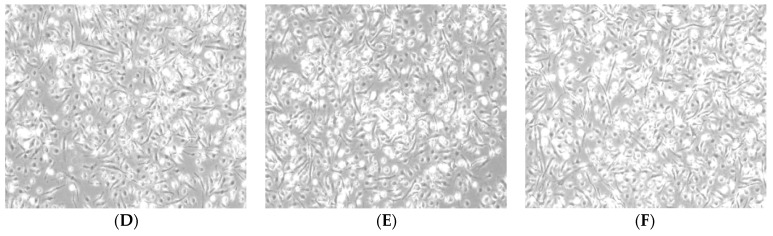
Morphology of BMDMs induced by IL-4 and/or RAP. (**A**) Control (normal BMDMs); (**B**) M2 macrophages (20 ng/mL of IL-4 induced BMDMs for 24 h); (**C**) M2 macrophages treated with 30 μg/mL of RAP; (**D**) M2 macrophages treated with 100 μg/mL of RAP; (**E**) M2 macrophages treated with 300 μg/mL of RAP; (**F**) M2 macrophages treated with 1 μg/mL of LPS.

There were no significant differences in the morphology of BMDMs induced by 20 ng/mL of IL-4 and that of BMDMs in the control group (Figure 2A,B). The M2 phenotype BMDMs treated with 100 μg/mL and 300 μg/mL of RAP appeared sharper, larger, and longer than those of the control cells. Furthermore, the morphology of the M1 phenotype BMDMs induced by RAP is similar to the morphology of the BMDMs induced by 1 μg/mL of LPS (Figure 2E,F).

### 2.3. Analysis of M1 Marker Expression on BMDMs’ Surface

**Figure 3:**
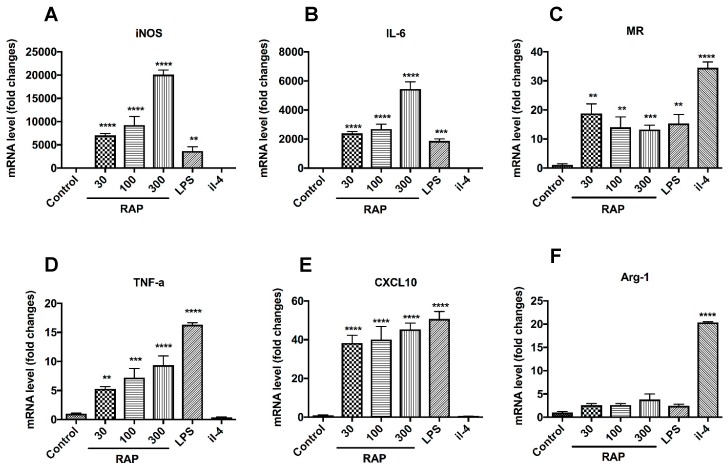
The expression of M1 and M2 markers in BMDMs induced by RAP and IL-4 was evaluated by qRT–PCR. BMDMs were treated with 30 μg/mL, 100 μg/mL, and 300 μg/mL of RAP, 1 μg/mL of LPS, and 20 ng/mL of IL-4 for 24 h. Total RNA was extracted and the mRNA levels of iNOS (**A**), IL-6 (**B**), MR (**C**), TNF-α (**D**), CXCL10 (**E**), and Arg1 (**F**), were determined by qRT-PCR. Data represent fold induction of mRNA expression compared with control group. A significant difference of each gene expression in each group was observed by one-way ANOVA test, **** *p* < 0.0001, *** *p* < 0.001, ** *p* < 0.01.

To evaluate the phenotype of BMDMs induced by RAP and LPS, we next investigated the representative gene expression of M1 and M2 phenotype by qRT-PCR (Figure 3). mRNA of iNOS (Figure 3A), IL-6 (Figure 3B), TNF-a (Figure 3D), and C-X-C motif chemokine 10 (CXCL10) (Figure 3E) were significantly induced in the RAP and LPS treatment group compared with the control group. In contrast, mRNA expression of mannose receptor (MR) and Arginase 1 which are typical M2 markers, was higher in the IL-4 treatment group compared with the RAP alone and LPS alone treatment groups (Figure 3C,F).

**Figure 4:**
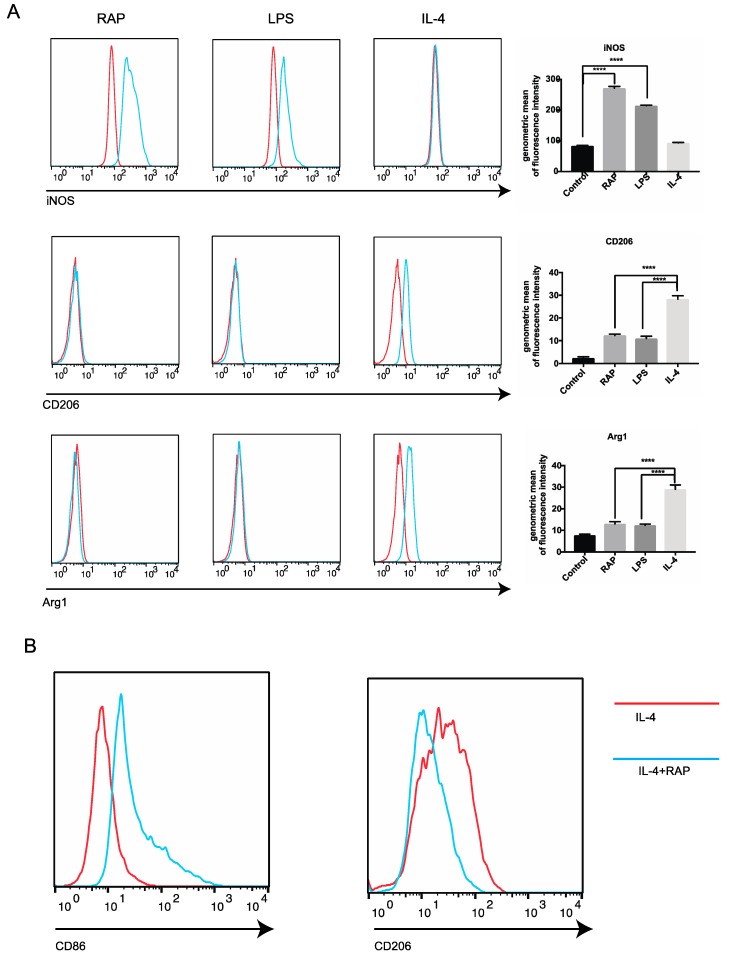
Analysis of expression of surface molecules on BMDMs by FCM. The BMDMs were stimulated with RAP, LPS and IL-4 for 24 h, then iNOS, CD206, and Arg1 expression level on BMDMs was determined by FCM (**A**). The statistical analysis of the expression of membranous molecules in the RAP group versus that in the control group was showed using Graphpad Prime. Columns, the percentage of the mean of three replicates; bars, SD (**A**). CD86 and CD206 expression on BMDMs stimulated with IL-4 or IL-4 plus RAP was analyzed by FCM (**B**). A significant difference of each gene expression in each group was observed by one-way ANOVA test, **** *p* < 0.0001.

FCM analysis was consistent with these findings. RAP induced iNOS expression, but IL-4 did not. Arginase 1 and CD206 expression were increased when BMDMs were induced by 20 ng/ mL of IL-4 for 24 h. However, RAP did not induce Arginase 1 and CD206 expression on BMDMs (Figure 4A). After 20 ng/mL of IL-4 stimulation for 24 h, CD206 expression on BMDMs was increased, and the BMDMs showed M2 phenotype (Figure 4B). However, the CD206 expression on BMDMs was decreased by RAP treatment (Figure 4B). The M1 marker CD86 was increased in BMDMs of the RAP-treated group. All of this evidence leads to the conclusion that RAP-induced BMDMs were polarized to M1 phenotypes.

### 2.4. Gene Expression of the Notch Signaling Pathway Induced by RAP

**Figure 5:**
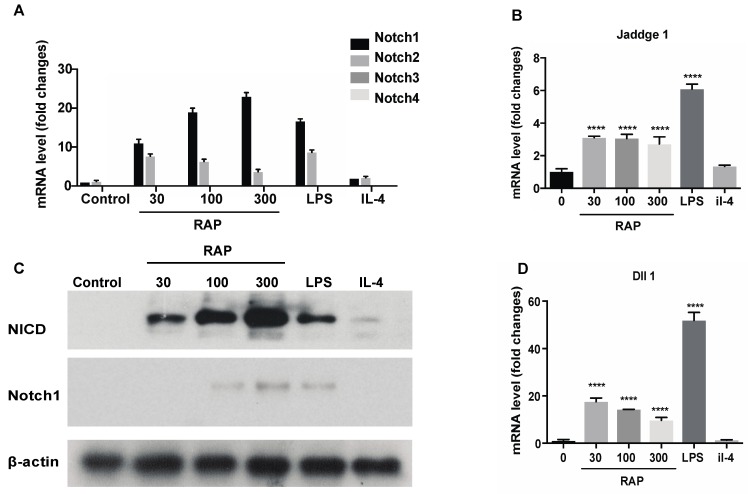
Gene expression of Notch signaling pathway induced by RAP. The RAW264.7 cells were stimulated with 30 μg/mL, 100 μg/mL, and 300 μg/mL of RAP, 1 μg/mL of LPS, and 20 ng/mL of IL-4 for 24 h. Gene expression levels of Notch1, Notch2, Notch3, Notch4 (**A**), Jaddge 1 (**B**), Dll1 (**D**) were measured by real-time PCR and normalized to that of β-actin. Values are shown as the fold-change relative to the control group. The NICD and Notch1 expression was confirmed by western blot (**C**), and β-actin was used as the control. A significant difference of each gene expression in each group was observed by one-way ANOVA test, **** *p* < 0.0001.

To investigate the molecular mechanism of how RAP affects BMDMs, expression of Notch receptors was investigated using qRT-PCR. Our previous study showed that the NO, IL-6 and TNF-α production in the supernatant of the macrophage-like cell line RAW264.7 cells induced by RAP was highly elevated [16]. This result suggested that the macrophage-like cell line RAW264.7 cells also showed M1 polarization when they were exposed to RAP. Thus, RAW264.7 cells were used for the molecular mechanism study. RAW264.7 cells were treated with 30 μg/mL, 100 μg/mL, and 300 μg/mL of RAP, 1 μg/mL of LPS, and 20 ng/mL of IL-4 for 24 h. Then, the expression of Notch1, Notch2, Notch3, and Notch4 receptors was analyzed by qRT-PCR. As shown in Figure 5A, stimulation with RAP and LPS for 24 h significantly triggered up-regulation of Notch1 and Notch2 transcription in a dose-dependent manner, showing that RAP and LPS are strong inducers. Functional diversity and functional redundancy among these four receptors have been reported [30,31]. The expression of Notch3 and Notch4 was not affected by stimulation. The up-regulation of Notch1 and NICD were also confirmed by Western blot in Figure 5C.

In addition, we examined the expression of the Notch target genes, Jaddge1 and Dll1, as markers for activation of Notch signaling. As shown in Figure 5B,D, treatment of RAW264.7 cells by LPS and RAP, Jaddge1 and Dll1 expression was up-regulated. Taken together, the evidence suggests that treatment with LPS and RAP stimulates Notch receptor expression in the macrophage-like cell line RAW264.7, and this stimulation triggers activation of Notch signaling. SOCS3 (this gene controls M1 and M2 polarization by Notch signaling [25]) expression in RAP-treatment group was increased as compared with that of the control group and IL-4 treatment group (Supplementary Information Figure S3).

**Figure 6:**
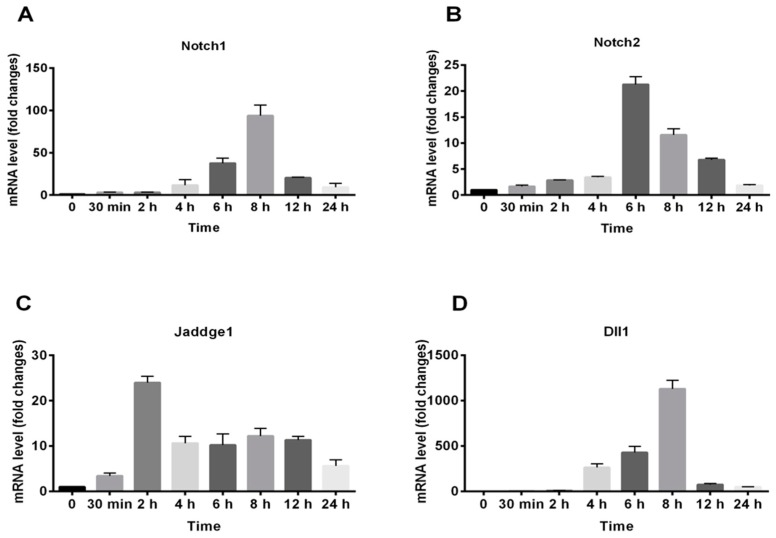
Gene expression profiles of Notch1, Notch2, Jaddge 1, and Dll1 in RAP-activated RAW264.7 macrophage cell lines. RAW264.7 cells were treated with RAP (100 μg/mL) with different time. The total RNA was isolated at indicated times. Expressions of Notch1 (**A**), Notch 2 (**B**), Jaddge 1 (**C**), and Dll1 (**D**) were analyzed by qRT-PCR.

For further determining the exact time of gene expression, RAW264.7 cells were treated with 100 μg/mL of RAP and RNA was isolated at specific times as indicated in Figure 6. Then each gene expression was determined by qRT-PCR. As shown in Figure 6, the time point of gene expression peak of Notch 1, Notch 2, and Dll 1 were all about 6 h to 8 h, while the time point of Jaddge 1 expression peak was 2 h. The gene expression of Notch 1, Notch 2, and Jaddge 1 was up-regulated as early as 30 min after RAP treatment. However, Dll 1 gene expression was not increased by RAP at 30 min.

### 2.5. Blocking of the Notch Signaling Pathway Results in M1 Marker Decrease even in the Presence of RAP

**Figure 7:**
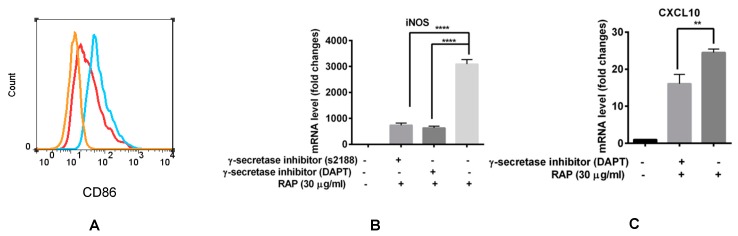
Blocking of Notch signaling pathway by γ-secretase inhibitor decreased M1 marker expression in the presence of RAP. (**A**) The CD86 expression was analyzed by FCM. The orange line represents the control cells. The blue line represents the RAW264.7 cells that were treated with 100 μg/mL of RAP. The red line represents the RAW264.7 cells that were pre-treated with s2188 (80 μM) for 1 h, and then treated with 100 μg/mL of RAP. (**B**) RAW264.7 cells were pre-incubated with s2188 (80 μM) and DAPT (80 μM) for 1 h, and then treated with 30 μg/mL of RAP. (**C**) RAW264.7 cells were pre-incubated with DAPT (80 μM) for 1 h, and then treated with 30 μg/mL of RAP. RNA expression of iNOS and CXCL10 were tested by qRT-PCR. A significant difference of each gene expression in each group was observed by one-way ANOVA test, **** *p* < 0.0001, ** *p* < 0.01.

For further investigated the exact role of Notch signaling in macrophage polarization induced by RAP, γ-secretase inhibitor that abrogates Notch signaling was used in our experiment. The results showed that when Notch signaling was blocked by s2188 and DAPT, iNOS expression was significantly decreased even in the presence of 30 μg/mL of RAP (Figure 7B). CXCL10 expression was also decreased by DAPT (Figure 7C). The γ-secretase inhibitor s2188 did not inhibit CXCL10 expression in RAP-induced RAW264.7 cells (data not shown); however, it did inhibit CD86 expression in RAP-induced RAW264.7 cells (Figure 7A). All these results suggest that the Notch signaling pathway participates in the RAP-induced macrophage polarization.

## 3. Materials and Methods

### 3.1. Materials

#### 3.1.1. Materials

APC anti-F4/80, FITC iNOS type II, and mouse arginase 1 fluorescein-conjugated antibody were purchased from eBioscience, BD Biosciences, and R&D System, respectively. FITC anti-CD206 and PE anti-CD86 were bought from BioLegend (San Diego, CA, USA) SYBR^®^ Select Master Mix for qRT-PCR amplification was purchased from Invitrogen Life Technologies (Carlsbad, CA, USA). Monoclonal anti-Notch1, monoclonal cleaved Notch1, **β**-actin, anti-rabbit IgG HRP-linked antibody and anti-mouse IgG HRP-linked antibody were obtained from Cell Signaling Technology Inc. (Beverly, MA, USA). Primers were purchased from Invitrogen Life Technologies (Carlsbad, CA, USA). γ-secretase inhibitors (DAPT and s2188) were purchased from Sigma-Aldrich (St. Louis, MO, USA).

RAP was isolated from boiling water extract of Astragali Radix, followed by leaching, ethanol precipitation, dialysis, protein depletion, combination, and concentration. RAP used in this study is same to the one we used in our pervious study [16]. The endotoxin of RAP samples was detected by PYROGENT™ Plus gel clot LAL assay kit (Lonza, Cologne, Germany) according to the manufacturer’s instruction. No endotoxin contamination was found in RAP.

#### 3.1.2. Cells Cultures and Animals

Inbred strain BALB/c mice (approximately 6–8 week-old, female) were purchased from the Laboratory Animal Services Centre of The Chinese University of Hong Kong (Hong Kong, China). The animals were provided with standard pellet diet and water ad libitum and maintained under controlled conditions of temperature and humidity, with 12 h light/dark cycles.

This study was carried out in accordance with the principles of the Basel Declaration and recommendations of the European Community guidelines (Directive 2010/63/EU). The protocol was approved by the Animals Ordinance, Department of Health, Hong Kong Special Administration Region, China ((15–33) in DH/HA & P/8/2/6 Pt.4).

The murine mammary carcinoma 4T1 cells and RAW264.7 cells were originally purchased from ATCC (American Type Culture Collection). 4T1 cells and RAW264.7 cells were cultured at 37 °C in a humidified 5% CO_2_ in RPMI 1640 and high glucose DMEM medium (Invitrogen Life Technologies, Carlsbad, CA, USA), respectively, containing 10% heat-inactivated FBS (Gibco, Carlsbad, CA, USA).

Bone marrow–derived macrophages (BMDMs) were prepared according to the protocol of Lake et al. [32,33]. Briefly, bone marrow cells were collected from the femoral shafts of female BALB/c mice. The red blood cells were lysed with red blood cells lysis buffer (Biolegend, CA, USA). The remaining cells were washed twice with PBS and re-suspended in RPMI1640 containing 10% heat-inactivated FBS. Then, they were cultured and differentiated for 7 days in RPMI1640 containing 10% heat-inactivated FBS and 10 ng/mL MCSF-conditioned medium. The cell monolayers were then treated overnight with IL-4 or LPS to induce differentiation into the M2 phenotype or M1 phenotype. To determine whether the macrophages had the M2 phenotype, the expression of macrophage CD206 was routinely estimated by FCM.

#### 3.1.3. Mouse Models

For the tumor-bearing model, BALB/c mice were divided into 2 experimental groups (*n* = 3). One group of mice was injected with 5 × 10^4^ of 4T1 cells in mammary gland fat pad. For the therapeutic model, all mice were injected with 5 × 10^4^ of 4T1 cells and 1 × 10^5^ of BMDMs stimulated with RAP in the same anatomic location. After injection, all the tumor volume for each mouse were determined by vernier caliper every 3 days. After 30 days’ measurements, the mice were euthanized and tumors were stripped to determine the tumor weight.

### 3.2. Analysis of Macrophage Surface Antigen Expression by Flow Cytometry

Surface antigen expression (F4/80, iNOS, arginase 1, CD86, and CD206) on BMDMs/Raw 264.7 cells induced by RAP or IL-4 were determined by FCM. BMDMs and RAW264.7 cells were disassociated and collected by pipetting repeat. The, cells were washed with PBS once and stained with the following monoclonal antibodies diluted in 1% FBS in PBS: APC anti-F4/80 (eBioscience), FITC iNOS type II (BD Biosciences), mouse arginase 1 fluorescein-conjugated antibody (R&D system), FITC anti-CD206 (BioLegend), PE anti-CD86 (BioLegend). For intracellular staining, the Foxp3 buffer set (eBioscience) was used; the cells were fixed and permeated for 30 min. After staining, the cells were washed twice with PBS and analyzed by BD Bioscience FACS Canto II flow cytometry (BD Biosciences). The data were analyzed using FlowJo software. The amount of surface antigen expression was calculated according to the previous study [34].

### 3.3. Cell Morphology

BMDMs were prepared as before (Part 2.1.2) at a density of 1 × 10^4^ cells/well in 24-well plate. Then the cells were treated with LPS, IL-4 or RAP in different concentrations. Each concentration was repeated in three wells. After being incubated with different medium for 24 h, cell morphology was observed and photographed using a 409 objective lens (Olympus BX-51, Tokyo, Japan). Ten fields were randomly selected under a microscope for each well.

### 3.4. RNA Isolation and qRT-PCR

BMDMs were harvested and total cellular RNA were extracted using Trizol Reagent (Invitrogen, Carlsbad, CA, USA). The RNA concentrations were determined by measuring the OD values. 2 μg of total RNA was used for the reverse transcriptions using SMARTer PCR cDNA synthesis kit (Takara, Japan). 1 μL of cDNA was amplified by PCR in a 20 μL reaction volume containing 10 μL GoTaq qPCR master mix (Promega, Madison, WI, USA) and 9 μL of H_2_O. The subsequent PCR amplification was performed using a ViiATM 7 Real-Time PCR System (Life Technologies, Carlsbad, CA, USA). Relative quantification of gene expression in treatment groups vs control group was calculated using the comparative threshold cycle method. M1 phenotype macrophages generally produce high levels of IL-6, NO, TNF-α and highly express CXCL10, while M2 phenotype macrophages have up-regulations of mannose receptor and arginine metabolism [18,19]. Thus, these markers are all selected in our present study to distinguish the M1 or M2 phenotype macrophages induced by different reagents. The primers used are listed in Supplementary Information Table S1.

### 3.5. Western Blot Analysis

Adherent macrophages were collected by centrifugation after digestion. Cell pellets were lysed using RIPA lysis buffer (pH 7.4) to obtain protein extracts. Homogenized samples were centrifuged at 13,000 rpm for 15 min at 4 °C and the resulting supernatants were saved. The amount of protein in the extracts was measured using Bio-Rad detergent-compatible protein reagent. 50 mg of total protein for each sample was subjected to 10% SDS polyacrylamide gel electrophoresis and then transferred to polyvinylidene difluoride (PVDF) membranes. The membranes were blocked in 5% powder milk of TBST. Three antibodies were used for incubating membranes overnight at 4 °C: anti-Notch-1; anti-NICD; and anti-β-actin. The membrane was incubated with secondary antibodies 1 h at room temperature. After washing, equal amounts of ECL (Bio-Rad, Hercules, CA, USA) were applied and exposed in a dark room for 45–120 s. The exposed film was scanned using an Epson scanner (Epson, Long Beach, CA, USA). All experiments were repeated in triplicate.

### 3.6. Statistical Analysis

Results were expressed as mean ± SD for at least three measurements. One-way ANOVA test (the un-treated group served as a control group) was used to determine the statistical significance. Statistical analyses were conducted using GraphPad Prism 5.0 software (GraphPad Software Inc., San Diego, CA, USA). Significant differences: *p* < 0.05.

## 4. Discussion

Activated macrophages can be roughly classified into two phenotypes, M1 and M2, based on their activities. In the tumor micro-environment, M1 phenotype macrophages suppress tumor growth, while M2 phenotype macrophages stimulate tumor growth. In general, M1 phenotype macrophages produce effector molecules, nitrogen intermediates, and inflammatory cytokines, IL-6, TNF-α and IL-1β. In contrast, the M2 phenotype macrophages have high levels of mannose receptors and arginine metabolism. Different phenotypes of macrophages express different sets of molecules and show different functional properties that are strongly dependent on the stimuli in the environment [35]. Our results showed that macrophages induced by RAP were the M1 phenotype because they significantly reduced tumor growth, represented by decreased tumor weight and volume. In addition, RAP promoted the M1 marker expression of macrophages, including iNOS, IL-6, TNF-α and CXCL10. Furthermore, RAP skewed macrophages from M2 to M1 phenotype, showing down-regulation of CD206 expression and up-regulation of CD86 expression.

SOCS family members are negative regulators of cytokine signals and thus play an important role in reducing cytokines’ production but also are directly induced by Toll-like receptors’ signaling pathways [36]. Our previous study showed that TLR4 participate in RAP-induced cytokine production in RAW264.7 cells [16]. Thus, the current result, that SOCS3 gene expression in macrophage cells is induced by RAP, is consistent with our previous study. Several reports have showed that higher expression of SOCS3 is uniquely associated with macrophage polarization (classic M1 macrophage activation) [37,38]. Most M1 phenotype macrophages in human and rodent animals showed up-regulation of SOCS3 and iNOS expression. SOCS3 expression in macrophages is induced by Notch1, and SOCS3 is one of the downstream proteins of Notch signaling. Our current results suggest that Notch signaling changes macrophage phenotype through SOCS3.

Several reports and unpublished data have shown that the stimulation of macrophages with pro-inflammation factors not only causes M1-type macrophage activation but also the up-regulation of Notch pathway molecules, leading to the activation of canonical Notch signaling [27]. Our results show that RAP transformed macrophages into the M1 phenotype and induced Notch ligand up-regulation. Therefore, M1 activation of macrophages is accompanied by the activation of Notch signaling. The M1 markers of macrophages were decreased even in the presence of RAP when they were blocked with γ-secretase inhibitor. Our results reported here indicate that Notch activation in macrophages promotes M1 polarization. Also, some reports have showed that NF-κB and MAPKs are involved in Notch signaling [28]. Our previous study showed that RAP induced NF-κB and MAPKs [16]. Therefore, RAP might trigger M1 polarization through a combination of transcription factors including Notch signaling, NF-κB signaling and MAPKs signaling. However, given the complexity of both macrophage populations and the Notch signaling pathway, more studies are needed to show the requirement of specific Notch pathway molecules in the polarization of specific subtype macrophages.

## 5. Conclusions

RAP-treated macrophages exhibit tumor suppressive effects and the anti-tumor M1 phenotype. Moreover, RAP is able to reverse macrophage polarization from M2 to M1. The Notch signaling pathway may play a critical role in M1 phenotype polarization for macrophage like cell line RAW264.7 cells induced by RAP.

## Supplementary Materials

The Supplementary Materials are available online.
