# Supplementary material for: Astragalus Polysaccharide RAP Induces Macrophage Phenotype Polarization to M1 via the Notch Signaling Pathway
Source: Molecules. 2019 May 27;24(10):2016. doi: 10.3390/molecules24102016 (PMC6572696; doi:10.3390/molecules24102016)
Supplement: Supplementary file 1 [file molecules-24-02016-s001.pdf]

Supplementary Materials: *Astragalus* Polysaccharide RAP Induces Macrophage Phenotype Polarization to M1 via Notch Signaling Pathway

Wei Wei, Zhi-Peng Li, Zhao-Xiang Bian and Quan-Bin Han

Table S1. Primers used in RT-PCR assay.

Gene name		Primers
TNF-α	forward	5′-ATGAGCACAGAAAGCATGATC-3′
	reverse	5′-TACAGGCTTGTCACTCGAATT-3′
IL-6	forward	5′-GATGCTACCAAACTGGATATAATC-3′
	reverse	5′-GGTCCTTAGCCACTCCTTCTGTG-3′
iNOS	forward	5′-GCCGTGGCCAACATGCTACT-3′
	reverse	5′-GGTCTTCCTGGGCTCGATCTG-3′
CXCL10	forward	5′-CCAAGTGCTGCCGTCATTTTC-3′
	reverse	5′-GGCTCGCAGGGATGATTTCAA-3′
Mannose receptor	forward	5′-CAAGGAAGGTTGGCATTTGT-3′
	reverse	5′-CCTTTCAGTCCTTTGCAAGC-3′
Arginase 1	forward	5′-TGGCTTGCGAGACGTAGAC-3′
	reverse	5′-GCTCAGGTGAATCGGCCTTTT-3′
Notch 1	forward	5′-AGAATGGCATGGTGCCCAG-3′
	reverse	5′-TGGTGGAGAGGCTGCTGTGTAG-3′
Notch 2	forward	5′-GATGGAGGTGACTGTTCCCTCA-3′
	reverse	5′-CGTCTTGCTATTCCTCTGGCAC-3′
Notch 3	forward	5′-GATTTCCCATACCCACTTCGG-3′
	reverse	5′-TGTGTAATGCAAAACCCTCAGG-3′
Notch 4	forward	5′-GTTGAAGAATTGATCGCAGCC-3′
	reverse	5′-AGGAAAAGCGGCGTCTGTT-3′
Jaddge 1	forward	5′-AGAAGTCAGAGTTCAGAGGCGTCC-3′
	reverse	5′-AGTAGAGGCTGTCACCAAGCAAC-3′
Dll 1	forward	5′-GGACCTCAGTGAGAGGCATATGG-3′
	reverse	5′-GGCAATTGGCTAGGTTGTTCATG-3′
β-actin	forward	5′-TGTCCACCTTCCAGCAGATGT-3′
	reverse	5′-AGCTCAGTAACAGTCCGCCTAGA-3′

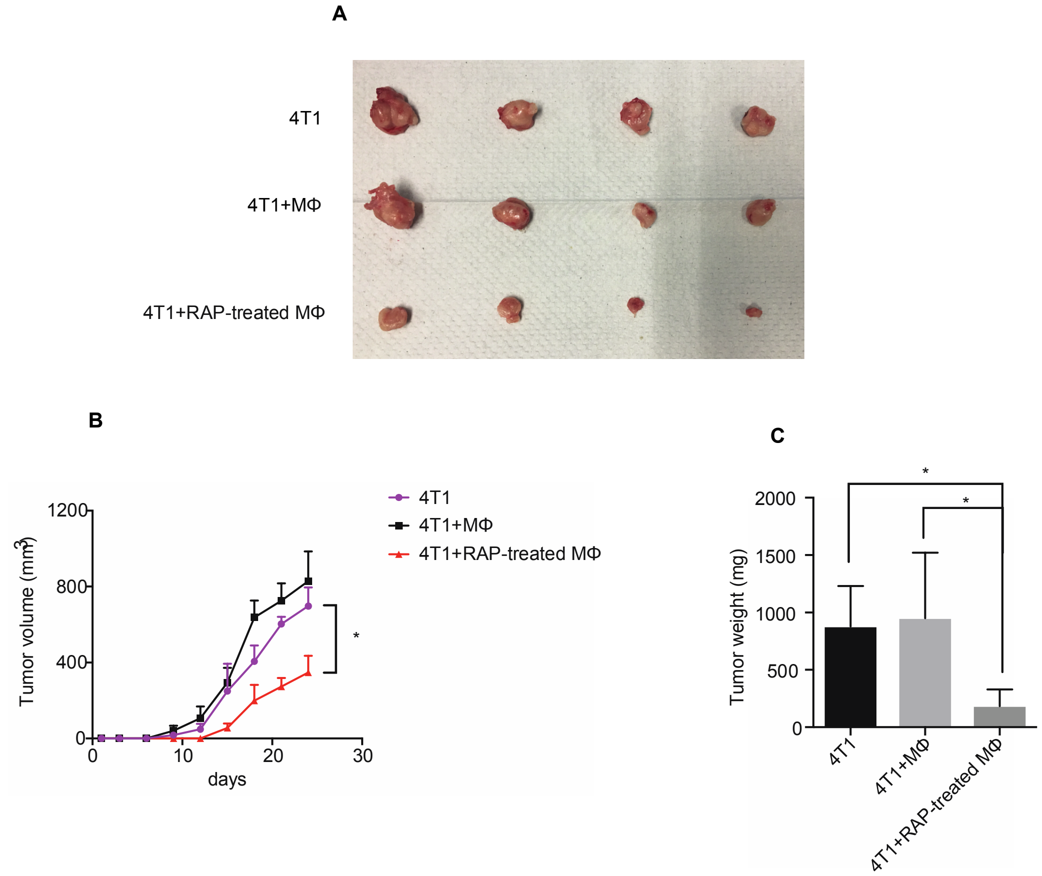

Figure S1. Tumoricidal effect of RAP-stimulated BMDMs in 4T1-induced animal model.

The BALB/c mice (4 mice/group) were injected with 4T1 cells, 4T1 cells plus BMDMs, and 4T1 cells plus RAP-treated BMDMs in mammary grand fat pad. Tumor volumes were measured every 3 days (B), and tumor weight were determined after 26 days. A significant difference of tumor weight was detected by one way ANOVA test (p<0.05) (A and C).

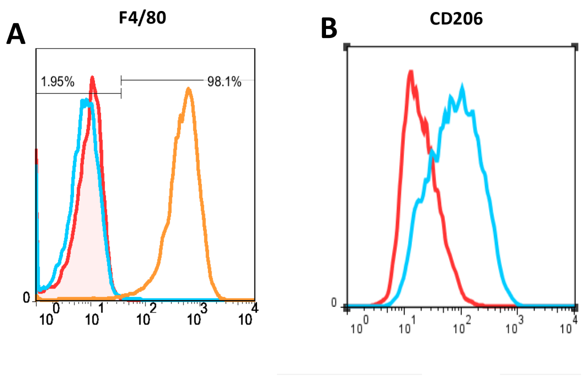

Figure S2. Histogram of F4/80 and CD206 fluorescent intensity of BMDMs induced by MCSF and IL-4 was analyzed by FCM.
(A) The bone marrow cells were treated with or without 10 ng/mL of MCSF for 7 days. Then the cells were stained with APC anti-F4/80 antibody and analyzed with FCM. The red line represents the isotype control. The orange line represents the bone marrow cells treated with 10 ng/mL of MCSF for 7 days. (B) BMDMs treated with or without 20 ng/mL of IL-4 for 24 h were stained with

FITC anti-CD206 antibody and analyzed with FCM. The red line represents the BMDMs. The blue line represents the BMDMs treated with 20 ng/mL of IL-4 for 24 h.

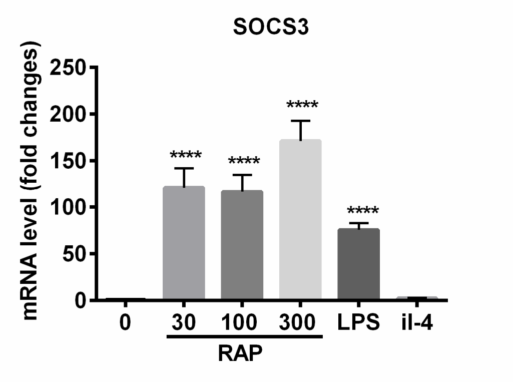

Figure S3. SOCS3 gene expression induced by RAP.
